# Note on the Breathing-Movements in the Two Sexes, and on the Alleged Influence of Stays in Producing Pulmonary Consumption

**Published:** 1853-05

**Authors:** W. H. Walshe

**Affiliations:** Fellow of the Royal College of Physicians, London; Professor of Medicine and Clinical Medicine at University College, London; etc.


### PATHOLOGY AND PRACTICE OF MEDICINE.

*Note on the Breathing-Movements in the two Sexes, and on the alleged Influence of Stays in producing Pulmonary Consumption.* [Excerpt from a Clinical Report.] By W. H. WALSHE, M. D., Fellow of the Royal College of Physicians, London; Professor of Medicine and Clinical Medicine at University College, London; etc.—Here, then, gentlemen, is a woman, (laboring under dilatation of the heart, with probable tricuspid regurgitation, and old pericarditis, general bronchitis, congestion, and œdema of both lungs,) whose maximum thoracic respiration-movements are translated from the upper to the lower regions of the chest. Her breathing-play is inferior-costal and abdominal, instead of being infraclavicular. She breathes, under the influence of her complicated malady, as the healthy male, and may be said to be *unsexed, quoad respiration*, by her disease. Observe, however, that this perversion only holds in *calm* breathing; the moment she takes a *forced* inspiration, the infraclavicular regions rise abruptly, fully, and equably, (she is non-tuberculous, be it remembered,) after the type of health. In the state of forced respiration she breathes—at least in the present point of view—precisely as both sexes breathe when the contents of the thorax are sound.

What is the cause of this perverted condition of breathing-movement in the female, when laboring under certain thoracic diseases? A preparatory point to determine is, the *how*—and, if possible, the *why*—of the difference in the calm breathing-movements of the sexes in health. And to this preliminary question we will confine our inquiries to-day.

The healthy, calm breathing of the male is essentially effected by the descent of the arch of the diaphragm; the amount of abdominal is greater materially than of pectoral expansion-movement; and the former commences sensibly before the latter, which is, besides, confined almost exclusively to the lower ribs. The male action is inferior costo-abdominal. But is not the ordinary breathing of the female carried on by similar play of the diaphragm? Judging from outward appearances, no. In the female the abdominal expansion is almost null, and always slightly posterior in point of time to the upper costal; neither do the lower ribs move notably, whereas the clavicles and infraclavicular regions rise and fall with freedom. The male seems to the eye to breathe with the abdomen and lower ribs from the sixth downwards; the female with the upper third of the chest alone. These statements refer to adults only.

To adults only, I say; for it is yet a point *sub judice*, whether, and to what proportional extent, the discrepancy of adult life prevails in infancy and early youth. I have examined a considerable number of female children, aged between four and ten years, who had never worn stays, or any substitute for these, and found in them the predominant infraclavicular action of the adult. But the excess of upper movement is very positively less than among their seniors. On the other hand, Boerhaave, one of the earliest observers of the difference in the respiratory action of the sexes, speaks as though the boy and girl of "one year old" breathe as distinctively, the one with the abdomen, the other with the chest, as the full-grown man and woman. *Per contra*, Beau and Maissiat affirm, that in earliest infancy, and often up to the third year, the respiration is abdominal in the female as well as in the male. It has appeared to me, too, that in earliest youth, when the pectoral and ventral modes of breathing become obvious, the chest action in the female is more *general* than at a later period, and less limited to the upper regions. Age, then, does seem to me to exercise an influence upon, or to be connected with, the typical breathing of the sexes.

Social position exercises none; the washerwoman and the peeress breathe exactly alike.

The habit of forced breathing is not without modifying power on the calm action in both sexes. For instance, the extensive play of the upper regions in full-chested *soprani*, kept up in the exercise of their art for many hours daily, ends by increasing the amount of infraclavicular movement in ordinary conversational breathing. It has appeared to me, that, even in *tenor* singers, some perversion of the ordinary condition—some degree of unnatural infraclavicular movement—may be detected in calm respiration.

But what influence does dress exercise? Looking at an adult female, and remembering her habit of drawing in the lower ribs by apparatus more or less unyielding, the inference seems unavoidable, that the reason why a woman does not breathe like a man is, that her mode of dress mechanically obstructs phrenic play. Certain mischiefs entailed by tight lacing we see positively in displacement of the liver; in mis-shapement of it, so that its height is made to exceed more or less its breadth, (as ascertained the other day, for instance, in the body of E. Smith, University College Hospital Female Case-Book, Vol. IX., p. 130;) in alterations of its texture, so that true lobular substance is replaced to a greater or less depth by induration-matter functionally inert. We see them exhibited in displacement of the heart,—in narrowing of the lower intercostal spaces, etc. And if, from certain of the facts concerning age just passed in review, we are forced to the admission, that the activity of infraclavicular respiration-movement in the female is in the main designed by nature, and independent of extraneous influence, still I cannot help thinking that the great excess of that movement, and the limitation of thoracic play to the upper thorax in the civilized adult female, are due in no small measure to the use of unyielding cases interfering with inferior costal and phrenic action. The agricultural female laborer, who knows not stays, breathes more like a male than a town female. Besides, during sleep, the conditions of pectoral and ventral action in the female are much less strikingly different from those in the male than in the waking state; the waist is relieved for a time from constriction. And, further, the male and female dog breathe almost exactly alike, as do also the horse and mare; the action is abdominal and lower costal.

It would seem, then, that stays are in part productive of the peculiarity of adult female breathing, but certainly are not its sole cause. Boerhaave, and his commentator, Haller, however, holding that the sexual difference obtains from birth, looked upon the free upper costal action in the female as a pre-ordainment to meet the difficulties of pregnancy. "Nisi hanc," says Boerhaave, "in fœminâ diversitatem natura fecisset, gravidæ perpetuâ dyspnœâ laboravissent, æque ac viri hydropici." But it seems here to be forgotten, that if the illustration be sound, ascitic females ought to escape dyspnœa. The final cause of the difference in the sexes is of less interest, however, than the mechanism by which it is actually worked out; but of this, also, nothing is known. Haller ascribed the predominant costal action in females to the greater flexibility of their bones and cartilages (Op. Cit., pp. 98, 145.) The upper interspaces are relatively wider in the female, the lower in the male; but is this effect, or cause, or neither one nor the other?

I have just reminded you of some of the evil influences exercised by the use of tightly-laced, unyielding stays on the liver. Do they inflict mischief on the lungs? It appears to me this will altogether depend on the amount of constriction. If this be simply sufficient to transfer the maximum chest-play from the base to the apex of the thorax, (or, rather to magnify somewhat the breathing difference superiorly and inferiorly natural to the female,) I cannot very clearly descry what evil is to come to the lungs, especially if the stays be cut bias, and be formed of yielding material. If, on the other hand, rigid wood-work or metal plates be used to stiffen stays of which the main material is hard and cut straight, then it is conceivable, *à priori*, that serious evil may come to the lungs. Remember, however, the wide difference in the statical and dynamic mechanism of the thorax and abdomen, and you will feel at once that the fact of serious compression of the liver being produced by tight lacing gives no shadow of proof that the pulmonary organs must suffer to similar amount, or even in similar fashion. I know not, as matter of clinical experience, what the mode of disturbance is which constriction of the base of the chest actually and demonstrably entails on pulmonary action or pulmonary structure. But such ignorance as this is not commonly avowed; on the contrary, the mass of information on the point is held to be positive and of ominous, most ominous, quality. Dr. Copland, for instance, writes, in a recent and otherwise admirable article, that the use of stiff stays produces "ultimately a morbid state of the blood, *tubercular deposition, especially in the lungs*, hæmoptysis, anæmia, etc." But it may be fearlessly asserted, that neither Dr. Copland, nor any other man living, could prove that the abuse of stays produces the specific disease, tubercle. I look in vain for evidence of such power in the writings of those who most loudly proclaim its existence; declamatory passages, arguing *à priori* from a loose physiology to a yet looser pathology, are all I have ever succeeded in finding—at least, with one seeming exception—a seeming one only. Mr. Farr, in truth, speaks thus: "Thirty-one thousand and ninety English women died in one year of the incurable malady, consumption. Will not this impressive fact induce persons of rank and influence to set their countrywomen right in the article of dress, and lead them to abandon a practice which disfigures the body, strangles the chest, produces nervous or" [this "or" is probably the "etc." of Dr. Copland] "other disorders; and *has an unquestionable tendency to implant an incurable hectic malady in the frame.*" Strange, Mr. Farr should forget to compare the relative mortality of the sexes in elucidating this question. Look at this table giving the mortality from consumption in three years to a million living of each sex in England and Wales :

| YEARS. | Deaths from Phthisis to 1,000,000 living of each Sex. | |
|---|---|---|
| | Males. | Females. |
| 1837 | 3,771 | 4,155 |
| 1838 | 3,783 | 4,077 |
| 1839 | 3,722 | 4,015 |

What evidence does this table give of the dependence of tuberculization on stays ? It simply shows that the phthisical mortality of females is somewhere about 300 per 1,000,000 living greater than that of males. Granting that the female excess is really due to stays, what scientific justification does its amount give of Mr. Farr's startling phrases ? None, absolutely none. But let me assure you, no particle of evidence exists that the moderate excess of female destruction is really traceable to the abuse of stays. Not a few arguments might be adduced, tending to prove their absolute innocence. Thus, in France, as is well known, females rarely use stays until the afternoon ; in England, women tighten themselves up the moment they rise in the morning; yet the excess of female phthisical mortality over the male is greater in France than in this country. Again, in certain parts of Europe, the men tighten themselves at the base of the chest, so as to produce a tolerably fair image of the figure of a wasp, and yet they do not seem thereby to increase their relative quota of phthisical mortality. Further, it will be conceded, that tight lacing is, as a rule, pushed to greater lengths among metropolitan than among rural female populations ; so that, if the influence assigned to stays be other than a figment of the brain, the plus destruction of women over males ought to be relatively greater in London than in the country. Now, such evidence as I can get at tells in precisely the contrary direction. Thus, examine these figures :

*Kent County.*

| | Males. | Females. |
|---|---|---|
| Population in 1841 - - - - - - | 232,228 | 236,885 |
| Absolute deaths from Phthisis - - - | 726 | 778 |
| Deaths from Phthisis per 1,000,000 living - | 3,126 | 3,242 |

*Metropolis.*

| Year. | Males. | Females. |
|---|---|---|
| Population in 1838 - - - - - - | 913,077 | 971,767 |
| Absolute deaths from Phthisis - - - | 4,057 | 3,630 |
| Deaths from Phthisis per 1,000,000 living - | 4,443 | 3,735 |

So that, actually, where, by fair inference, the amount of stay-constriction is greatest, and its prevalence widest, (in the Metropolis,) females are destroyed by phthisis to a less degree than males; whereas, amid a country population, which we may honestly assume to undergo a less mean amount of tightening, females die consumptive in notably larger proportion than males.

No, gentlemen, if the abuse of stays produces consumption, its power to do so most indubitably remains to be proved; and while the laws of an enlightened pathology point to the excessive improbability of an essentially diathetic disease springing from a mechanical cause, I entreat you not to adopt the popular creed, that "stays cause consumption," unless on direct and unimpeachable logical evidence. There is quite enough in the demonstrable evils entailed by tight lacing to justify you in warring against the abuse; you have no need to support your arguments by the unfair appeal to an imaginary mischief.—*London Med. Times and Gaz.*

